# Outcomes with single-agent gilteritinib for relapsed or refractory *FLT3*-mutant AML after contemporary induction therapy

**DOI:** 10.1182/bloodadvances.2024014017

**Published:** 2024-09-16

**Authors:** Jad Othman, Angela Hwang, Maximillian Brodermann, Islam Abdallah, Kayleigh McCloskey, Paolo Gallipoli, Georgina Clarke, Raymond Dang, Jennifer Vidler, Pramila Krishnamurthy, Faisal Basheer, Anne-Louise Latif, Renuka Palanicawandar, Tom Taylor, Asra Khan, Victoria Campbell, Francesca Hogan, Alex Kanellopoulos, Kathryn Fleming, Angela Collins, Chris Dalley, Justin Loke, Scott Marshall, David Taussig, Sreetharan Munisamy, Eleana Loizou, Heba Yassin, Mike Dennis, Rui Zhao, Edward Belsham, Duncan Murray, Nicole Fowler, Jenny O'Nions, Anjum Khan, Rob Sellar, Richard Dillon

**Affiliations:** 1 Department of Medical and Molecular Genetics, King's College London, London, United Kingdom; 2 Department of Haematology, Guy's and St Thomas' NHS Foundation Trust, London, United Kingdom; 3 Faculty of Medicine and Health, University of Sydney, Sydney, Australia; 4 Haematology, University College London Hospital NHS Foundation Trust, London, United Kingdom; 5 Department of Haematology, Leeds Teaching Hospitals Trust, Leeds, United Kingdom; 6 Haematology, St Bartholomew's Hospital, Barts Health NHS Trust, London, United Kingdom; 7 Barts Cancer Institute, Queen Mary University of London, London, United Kingdom; 8 Clinical Haematology, James Cook University Hospital, Middlesbrough, United Kingdom; 9 Haematology, King’s College Hospital, London, United Kingdom; 10 Haematology, Addenbrooke's Hospital, Cambridge, United Kingdom; 11 Department of Haematology, Queen Elizabeth University Hospital, Glasgow, United Kingdom; 12 Haematology, Imperial College Healthcare NHS Trust, Hammersmith Hospital, London, United Kingdom; 13 Department of Haematology, Nottingham University Hospital, Nottingham, United Kingdom; 14 Department of Clinical Haematology, Blackpool Teaching Hospitals NHS Foundation Trust, Blackpool, United Kingdom; 15 Haematology Centre, Western General Hospital, Edinburgh, United Kingdom; 16 Haematology Department, University Hospital of Wales, Cardiff, United Kingdom; 17 Clinical Haematology, Sheffield Teaching Hospitals NHS Trust, Royal Hallamshire Hospital, Sheffield, United Kingdom; 18 Bristol Haematology and Oncology Centre, University Hospital Bristol and Weston, Bristol, United Kingdom; 19 Haematology Department, Norfolk and Norwich University Hospitals NHS Foundation Trust, Norwich, United Kingdom; 20 Haematology Department, University Hospital Southampton, Southampton, United Kingdom; 21 Centre for Clinical Haematology, Queen Elizabeth Hospital, University Hospitals Birmingham NHS Foundation Trust, Birmingham, United Kingdom; 22 Haematology, City Hospitals Sunderland NHS Trust, Sunderland, United Kingdom; 23 Haemato-oncology Unit, Royal Marsden Hospital, The Royal Marsden NHS Foundation Trust, Surrey, United Kingdom; 24 Haematology Department, East Kent Hospitals University NHS Foundation, Canterbury, United Kingdom; 25 Haematology, Mersey and West Lancashire Teaching Hospitals NHS Trust, Whiston, United Kingdom; 26 Haematology, University Hospitals Sussex NHS Foundation Trust, Worthing, United Kingdom; 27 Haematology and Transplant Unit, The Christie NHS Foundation Trust, Manchester, United Kingdom; 28 Haematology, Torbay Hospital, Torquay, United Kingdom; 29 Haematology, Portsmouth Hospitals University NHS Trust, Portsmouth, United Kingdom; 30 Haematology, University Hospitals Coventry and Warwickshire NHS Trust, Coventry, United Kingdom; 31 Haematology, Royal Cornwall Hospitals NHS Trust, Truro, United Kingdom; 32 Department of Haematology, UCL Cancer Institute, London, United Kingdom

## Abstract

Gilteritinib is the current standard of care for relapsed or refractory fms related receptor tyrosine kinase 3 (*FLT3*)–mutated acute myeloid leukemia in many countries, however outcomes for patients relapsing after contemporary first-line therapies (intensive chemotherapy with midostaurin, or nonintensive chemotherapy with venetoclax) are uncertain. Moreover, reported data on toxicity and health care resource use is limited. Here, we describe a large real-world cohort of 152 patients receiving single-agent gilteritinib in 38 UK hospitals. Median age was 61 years, and 36% had received ≥2 prior lines of therapy, including a FLT3 inhibitor in 41% and venetoclax in 24%. A median of 4 cycles of gilteritinib were administered, with 56% of patients requiring hospitalization in the first cycle (median, 10 days). Over half of patients required transfusion in each of the first 4 cycles. Complete remission (CR) was achieved in 21%, and CR with incomplete recovery (CRi) in a further 9%. Remission rates were lower for patients with *FLT3*–tyrosine kinase domain or adverse karyotype. Day-30 and day-60 mortality were 1% and 10.6%, respectively, and median overall survival was 9.5 months. On multivariable analysis, increasing age, *KMT2A* rearrangement, and complex karyotype were associated with worse survival whereas *RUNX1* mutations were associated with improved survival. Twenty patients received gilteritinib as first salvage having progressed after first-line therapy with venetoclax, with CR/CRi achieved in 25% and median survival 4.5 months. Real-world results with gilteritinib mirror those seen in the clinical trials, but outcomes remain suboptimal, with more effective strategies needed.

## Key Points

• Outcomes with single-agent gilteritinib across 38 National Health Service hospitals mirror those seen in clinical trials.
• Patients with adverse karyotype, and those treated after venetoclax-based therapy had poor outcomes.

## Visual Abstract

**Figure:**
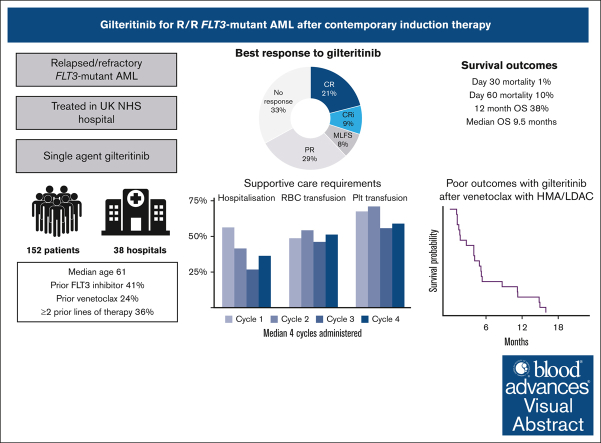

## Introduction

Somatic mutations in the gene encoding the fms-related receptor tyrosine kinase 3 (*FLT3*) are frequent in acute myeloid leukemia (AML).^1^ They are enriched in patients with normal karyotype and are associated with clinically aggressive disease characterized by leukocytosis and a high proportion of bone marrow blasts.^2^, ^3^, ^4^ The most common mutations are internal tandem duplications (ITD) in exons 14 to 15 (encoding the juxtamembrane domain) and point mutations in exon 20 (encoding the tyrosine kinase domain [TKD]). Patients with *FLT3-*ITD have a high rate of relapse and reduced overall survival (OS).^3^^,^^5^ Despite the incorporation of FLT3 inhibitors into first-line therapy, between one-third and one-half of patients with *FLT3* mutation suffer disease relapse.^6^^,^^7^

Outcomes for patients with relapsed or refractory *FLT3*-mutated AML remain unsatisfactory. The current standard of care in most countries is single-agent gilteritinib, which improved survival compared with salvage chemotherapy in the ADMIRAL study.^8^ In this study, the median OS was 9.3 months with gilteritinib compared with 5.6 months with chemotherapy, and gilteritinib was associated with increased rates of remission and allogeneic stem cell transplant. However, very few patients in the ADMIRAL study had received prior treatment with FLT3 inhibitors (49/371, 13%) or venetoclax (none).^9^ This is important because the standard of care for both younger and older patients has changed since the ADMIRAL study was performed. In most parts of the world, the current first-line standard-of-care therapy for younger patients with *FLT3*-mutant AML is cytarabine and an anthracycline combined with a FLT3 inhibitor (midostaurin or quizartinib), whereas older patients now usually receive venetoclax with azacitidine.^10^ The outcomes for these patients treated with gilteritinib at relapse are therefore uncertain. Furthermore, the ADMIRAL study had relatively restrictive inclusion criteria (eg, being limited to patients with 1 prior line of therapy) and did not report detailed data on the use of health care resources (such as hospital admission and blood products). To date corroborative real-world data have also been relatively limited.^11^, ^12^, ^13^

Early phase studies of FLT3 inhibitor-containing combination therapies at relapse have shown promising preliminary data,^14^^,^^15^ however the interpretation of these studies is limited by the lack of accurate data on response rates, toxicity, health care resource use and long-term outcomes with single-agent gilteritinib in patients receiving contemporary first-line therapies. To address this, we performed a retrospective analysis of a large cohort of patients with relapsed or refractory FLT3-mutated AML treated with single-agent gilteritinib in 38 centers across the UK National Health Service (NHS).

## Methods

### Patients

Patients were included in this study if they had relapsed or refractory *FLT3*-mutated AML and were treated with single-agent gilteritinib. Those treated for molecular relapse have been previously reported and were excluded from this analysis.^16^ Sites were invited to participate by an email from the study team, as part of a wider analysis of outcomes of novel agents for AML within the NHS. Participating centers were asked to include all patients treated at their site during the data collection period. Data were collected retrospectively by clinicians or research staff, anonymized and entered into a central REDCap database. Gilteritinib dose, duration, and toxicity information was requested for the first 4 cycles of therapy. After an initial phase performed as a service evaluation in July 2021, subsequent data were collected as part of a project approved by the Central Bristol Research Ethics Committee (22/SW/0042).

### Treatment

Gilteritinib was approved by the UK NHS as an emergency measure during the coronavirus pandemic in April 2020 and was formally approved by the National Institute for Health and Care Excellence in August 2020 for use in patients with relapsed or refractory *FLT3*-mutated AML. The recommended starting dose was 120 mg daily, with caution advised but no dose modification when used in combination with an azole antifungal. Dose modifications for toxicity or lack of response were at the discretion of the treating clinician.

### End points and statistical methods

Responses were defined as per European LeukemiaNet (ELN) 2017 criteria,^17^ and assigned by the treating clinician based on bone marrow biopsies performed as part of routine care. Responses after subsequent therapy or transplant were not considered. Median follow-up was computed using the reverse Kaplan-Meier method.^18^ OS was calculated from day 1 of cycle 1 until the day of death, censored on the date last known to be alive. Cumulative incidence of relapse was calculated for patients achieving complete remission (CR) or CR with incomplete hematological recovery (CRi), from the date of remission to the date of relapse or death, with nonrelapse mortality as a competing risk. Cumulative incidence of relapse was also calculated for patients achieving morphological leukemia-free state (MLFS) or partial remission (PR), from date of best response to documented disease progression.

Karyotyping, testing for *FLT3*-ITD, TKD, and *NPM1* mutations and next-generation sequencing (NGS) panels were performed at accredited local or regional laboratories, as deemed appropriate by the treating clinician. *FLT3* testing was repeated at relapse, whereas karyotype and NGS was performed only at diagnosis for most patients. If these were repeated at relapse, the most recent results were used to assign risk category. If karyotyping failed, this was considered to be intermediate risk for Medical Research Council (MRC) and ELN 2022 risk assignment. *NPM1* measurable residual disease (MRD) was performed by quantitative reverse transcription polymerase chain reaction at a central reference laboratory.^16^

Between-group comparisons were performed using Wilcoxon rank-sum test for continuous variables, χ^2^ test for categorical variables, and log-rank test for survival end points. Factors associated with OS were analyzed using Cox regression. Age was included in 10-year intervals, and genes with variants detected in at least 5% of patients were included. Missing data were not imputed. All analyses were performed with R statistical software version 4.3.2.

## Results

### Patient characteristics

A total of 152 patients were identified from 38 hospitals, with a median of 2 patients per hospital (range, 1-29; supplemental Figure 1). Median age was 61 years (range, 19-90; Table 1). Ninety-nine patients (65%) were treated for relapsed AML, the remainder were refractory to the last line of therapy. Prior therapy included intensive chemotherapy in 120 (79%), venetoclax in 37 (24%), and allogeneic transplant in 29 (19%). Sixty-three patients (41%) had received prior FLT3 inhibitors, most commonly midostaurin (58, 38%). Fifty-four patients (36%) had received ≥2 prior lines of therapy (range, 1-6). Compared with those treated in the second line, these patients were younger (median age, 56 vs 69) and more likely to have had first-line intensive therapy (87% vs 71%).

**Table 1.** **Baseline characteristics**

Patient characteristics	N = 152
Median age, y (range)	61 (19-90)
Female	72 (47%)
**Performance status**	
0-1	93 (82%)
≥2	21 (18%)
Missing	38
**Clinical disease type**	
De novo	120 (79%)
Secondary	27 (18%)
Therapy-related	5 (3.3%)
**Disease status**	
Refractory to last line of therapy	53 (35%)
Relapse	99 (65%)
**No. of prior lines of therapy (range)**	1-6
1	98 (64%)
≥2	54 (36%)
**Previous therapies**	
FLT3 inhibitor∗	63 (41%)
Midostaurin	58 (38%)
Quizartinib	3 (2.0%)
Sorafenib	7 (4.6%)
Intensive chemotherapy	121 (80%)
Venetoclax	37 (24%)
Allogeneic transplant	29 (19%)
**Intensity of first-line AML therapy**	
Intensive chemotherapy (DA, FLAG-Ida or CPX-351)	117 (77%)
Low intensity (azacitidine or LDAC with/without venetoclax)	35 (23%)
Venetoclax with azacitidine or LDAC	22 (14%)

DA, daunorubicin and cytarabine; FLAG-Ida, fludarabine, cytarabine, granulocyte-colony stimulating factor (G-CSF) and idarubicin; LDAC, low-dose cytarabine.

∗ Five patients previously exposed to both midostaurin and sorafenib.

Overall, 134 patients (88%) had *FLT3*-ITD at the time of gilteritinib treatment, 23 had *FLT3*-TKD, and 5 patients had both mutations (Table 2). In 21 patients (14%), the *FLT3* mutation was acquired at relapse, having been undetectable at diagnosis. This was much more common in patients previously exposed to venetoclax (12 of 37, 32%) than those not exposed (9 of 115, 8%). An *NPM1* comutation was present in 34% of patients. Of 125 patients with NGS results available, *DNMT3A* was the most frequently mutated gene (35%), followed by *RUNX1* (20%).

**Table 2.** **Disease characteristics**

Characteristic	N = 152
*FLT3*-ITD∗	134 (88%)
*FLT3*-TKD∗	23 (16%)
**Evolution of *FLT3* mutation**	
Present at diagnosis and relapse	131 (86%)
Emergent upon relapse	21 (14%)
**Cytogenetic/FISH abnormalities**	
Normal karyotype	89 (62%)
+8	16 (11%)
*KMT2A* rearrangement	4 (2.7%)
*MECOM* rearrangement	3 (2.1%)
Complex karyotype	5 (3.5%)
Other adverse, noncomplex (del5q, −7, del17p)	8 (5.6%)
Failed or missing	6
**MRC cytogenetic risk**	
Intermediate	134 (88%)
Adverse	18 (12%)
*NPM1* mutation	52 (34%)
**Molecular mutations (present in >5%)**	
*DNMT3A*	44 (35%)
*RUNX1*	25 (20%)
*TET2*	15 (12%)
*SRSF2*	13 (10%)
*IDH2*	13 (10%)
*ASXL1*	12 (9.6%)
*WT1*	12 (9.6%)
*NRAS*	10 (8.0%)
*SF3B1*	9 (7.2%)
*IDH1*	8 (6.4%)
Missing	27
**ELN 2022 risk classification**	
Favorable	5 (3.3%)
Intermediate	96 (64%)
Adverse	50 (33%)

FISH, fluorescence in situ hybridization; MRC, Medical Research Council.

∗ Five patients had both *FLT3*-ITD and *FLT3*-TKD

Patient characteristics were similar to those in the gilteritinib arm of the ADMIRAL study, apart from more patients with >1 prior line therapy (36% vs 0%), more patients with prior FLT3 inhibitor exposure (41% vs 14%), more with prior venetoclax (24% vs not reported, assumed to be 0%), and more *FLT3*-TKDs (16% vs 9.3%: supplemental Table 1).

### Therapy administered and supportive care requirements

**Figure 1.**
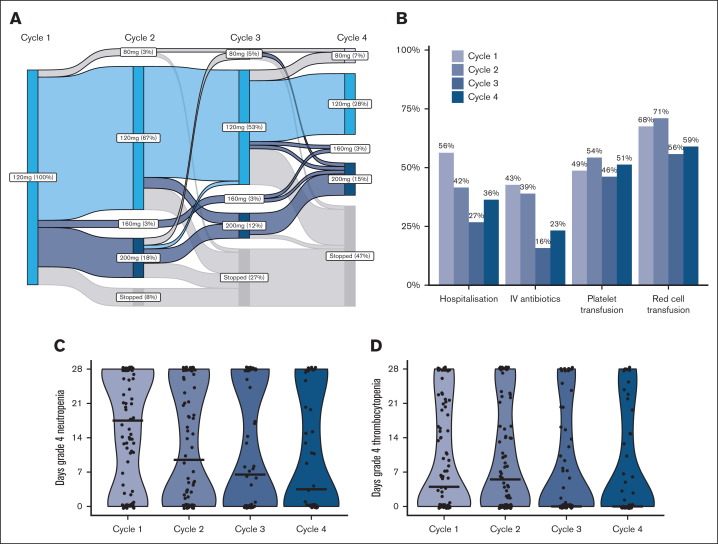
**Dose modifications, supportive care and cytopenias during cycles 1 to 4.** (A) Gilteritinib dose during cycles 1 to 4. (B) Proportion of patients requiring supportive care measures in cycles 1 to 4. (C) Days of grade 4 neutropenia in cycles 1 to 4. (D) Days of grade 4 thrombocytopenia in cycles 1 to 4.

A median of 4 cycles of gilteritinib was administered (range, 1-30), with therapy ongoing in 20 patients at time of data collection. Dose modifications were relatively frequent, usually dose increases in patients with suboptimal response (Figure 1A). Of 60 patients with data on gilteritinib dose in the first 4 cycles, 18 had a dose increase, for lack of response (n = 16) or relapse (n = 2), with improvement in response in 4 patients (22%).

Data on blood counts and supportive care were available for 88 patients. The median duration of grade 4 neutropenia was 18 days in cycle 1; decreasing to 10, 7, and 4 days in cycles 2, 3, and 4, respectively. Grade 4 thrombocytopenia lasted for a median of 4 days in cycle 1, 6 days in cycle 2, and 0 days in cycles 3 and 4 (supplemental Table 2). Overall, 56% of patients required hospitalization in the first cycle for a median of 10 days, whereas 68% required red blood cell transfusion and 38% platelet transfusion (Figure 1; supplemental Table 2). The need for hospitalization and intravenous antibiotics reduced over the first 4 cycles however transfusion requirements did not decrease.

### Response to therapy

**Figure 2.**
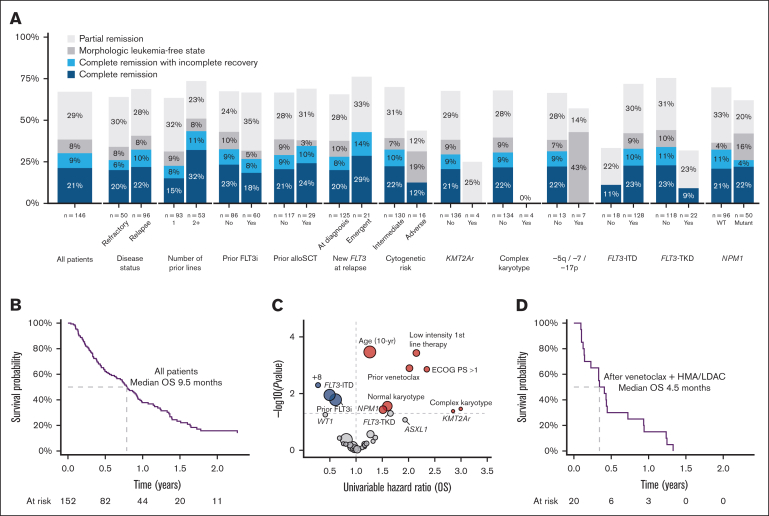
**Response rates and survival outcomes.** (A) Best response achieved by clinical and genomic subgroups. (B) Kaplan-Meier plot of OS, all patients. (C) Volcano plot of univariable HRs for OS. (D) Kaplan-Meier plot of OS for patients treated with gilteritinib as first salvage after venetoclax with low-dose cytarabine or azacitidine.

Remission status was documented in 146 patients, of whom 31 (21%) achieved CR and 13 (8.9%) CRi, for a composite CR (cCR) rate of 30%. A further 8.2% had MLFS and 29% PR (Table 3). In those achieving cCR, median time to first response was 32 days (95% confidence interval [CI], 28-56) and best response was 56 days (95% CI, 28-85). Responses were achieved in patients treated for both relapsed (32%) and refractory (26%) disease and were more frequent when treatment was at a later line of therapy (Figure 2). Prior FLT3 inhibitors or prior allogeneic transplant did not appear to affect the achievement of remission. Patients with *FLT3*-ITD had a higher rate of cCR (33%) than those with *FLT3*-TKD (9%). Patients with *FLT3*-TKD were more likely to receive gilteritinib as second-line therapy (78% vs 61% of those without *FLT3*-TKD), which may partly account for the lower response rate seen for those with only 1 prior line of therapy. Adverse cytogenetic risk, particularly complex karyotype, was associated with poor response rate (Figure 2).

**Table 3.** **Remission and outcome**

Characteristic	All patients, N = 152
Median number cycles gilteritinib (range)	4 (1-30)
**Best response**	
CR	31 (21%)
CRi	13 (8.9%)
MLFS	12 (8.2%)
PR	42 (29%)
Refractory disease	39 (27%)
Death before response assessment	9 (6.2%)
Missing	6
**Allogeneic transplant**	36 (24%)
Bridged directly to transplant with gilteritinib	16 (11%)
Transplant after additional therapy to deepen response	9 (5.9%)
Transplant after relapse/refractory disease	11 (7.2%)
**Survival**	
Day-30 mortality	1%
Day-60 mortality	10.6%
12-mo survival	38% (95% CI, 30-47)
Median survival (mo)	9.5 (95% CI, 7.7-11.3)

Patients achieving CR or CRi had 12-month cumulative incidence of relapse of 36%. For the 54 patients with best response of MLFS or PR, disease progression was frequent, occurring in 71% by 12 months (supplemental Figure 2).

Sixteen patients with *NPM1*-mutated AML had MRD responses assessed by *NPM1* quantitative reverse transcription polymerase chain reaction. Of 7 patients achieving CR or CRi, 1 achieved MRD negativity after 8 cycles, 1 achieved a >4-log reduction after 5 cycles, and the remainder had <1-log reduction. A further 4 patients had PR or MLFS and all had stable MRD levels (<1 log change). The other 5 patients had refractory disease, with a rise in MRD levels. No *FLT3* MRD testing was performed.

### Survival outcomes

Median follow-up was 21.4 months. Day-30 and day-60 mortality were 1% and 10.6%, respectively. Median OS was 9.5 months (95% CI, 7.7-11.3), with 38% of patients surviving to 12 months and 23% to 18 months (Figure 2). As expected, patients who had a documented response to therapy had better outcomes, with 12-month OS of 66% in patients achieving CR and 64% in those with CRi (supplemental Figure 3).

On univariable analysis, survival was better for patients with prior FLT3 inhibitor exposure, *FLT3*-ITD, and trisomy 8 but worse for older patients, those with performance status of ≥2, low-intensity first-line therapy, prior venetoclax exposure, normal karyotype, complex karyotype, *KMT2A* rearrangement, and *NPM1* mutation (Figure 2). Multivariable analysis demonstrated that age (hazard ratio [HR], 1.51; 95% CI, 1.21-1.89), *KMT2A* rearrangement (HR, 6.63; 95% CI, 1.46-30.2), and complex karyotype (HR, 5.71; 95% CI, 1.03-31.6) were associated with worse survival whereas *RUNX1* mutations (HR, 0.34; 95% CI, 0.14-0.79) were favorable (Table 4).

**Table 4.** **Multivariable regression for OS**

Characteristic	HR	95% CI	*P* value
Age (10-y increments)	**1.51**	**1.21-1.89**	**<.001**
**Clinical disease type (compared with de novo)**			
Secondary	0.53	0.26-1.08	.081
Therapy-related	2.49	0.69-8.99	.2
Relapsed disease (compared with refractory)	0.70	0.38-1.30	.3
≥2 prior lines of therapy	1.65	0.86-3.17	.13
Prior FLT3 inhibitor	0.87	0.49-1.55	.6
Low-intensity therapy in first line	1.73	0.75-3.95	.2
Prior allogeneic transplant	1.16	0.52-2.58	.7
Prior venetoclax	1.22	0.55-2.71	.6
Normal karyotype	1.53	0.63-3.72	.3
+8	0.60	0.17-2.11	.4
*KMT2A* rearrangement	**6.63**	**1.46**-**30.2**	**.014**
*MECOM* rearrangement	0.83	0.13-5.28	.8
Complex karyotype	**5.71**	**1.03**-**31.6**	**.046**
Other adverse cytogenetic abnormality	0.55	0.10-3.09	.5
*FLT3*-ITD	0.66	0.07-6.27	.7
*FLT3-*TKD	1.05	0.11-9.69	>.9
*NPM1* mutation	1.15	0.60-2.21	.7
*DNMT3A* mutation	0.84	0.43-1.65	.6
*ASXL1* mutation	1.94	0.71-5.31	.2
*RUNX1* mutation	**0.34**	**0.14**-**0.79**	**.013**
*SF3B1* mutation	1.15	0.31-4.36	.8
*SRSF2* mutation	1.89	0.71-5.08	.2
*IDH1/2* mutation	1.39	0.67-2.90	.4
*TET2* mutation	1.17	0.52-2.63	.7
*N/KRAS* mutation	0.55	0.18-1.69	.3
*WT1* mutation	0.99	0.35-2.84	>.9

Bold face denotes statistically significant variables

Given the survival benefit seen in ADMIRAL for gilteritinib in patients with combined *NPM1* and *DNMT3A* mutations,^8^^,^^19^ we examined outcomes by the combination of mutations in these 2 genes in patients treated for *FLT3*-ITD AML. Although the differences were not significant, we found that the proportion of patients achieving cCR and median survival were best for patients in whom both *NPM1* and *DNMT3A* were either mutant or wild-type, whereas those with *NPM1* mutation only appeared to have the worst outcomes (supplemental Table 3; supplemental Figure 4).

### Outcomes with transplant

Thirty-six patients (24%) underwent allogeneic transplant after treatment with gilteritinib, including 16 (11%) bridged directly with gilteritinib and a further 9 with response to gilteritinib but who had additional therapy (7 switched to chemotherapy and 2 venetoclax added to gilteritinib) to deepen response before transplant (Table 3). The remaining 11 patients did not respond to gilteritinib and received transplants after further lines of therapy for relapsed or refractory disease.

Of the 98 patients who achieved PR or better with gilteritinib, the 25 who proceeded to transplant were younger (median age, 44 vs 69 years), had better performance status and were more likely to have been treated for refractory disease (supplemental Table 4). The median survival from transplant was 1.17 years, with 52% alive at 1 year and 28% at 2 years after transplant (supplemental Figure 5). By time-dependent Cox regression, allogeneic transplant was associated with a HR for death of 0.61 (95% CI, 0.31-1.19).

### Gilteritinib as second-line therapy after venetoclax and azacitidine or low-dose cytarabine

Overall, 20 patients received gilteritinib as first salvage after front-line therapy with venetoclax and azacitidine or low-dose cytarabine. Median age was 72 years, and 35% were treated for refractory disease (supplemental Table 5). *FLT3* mutation was not present at diagnosis in 40% of patients. Five patients (25%) achieved cCR, and median OS was 4.5 months, with only 15% alive at 12 months and none at 18 months (Figure 2D). Median OS from the time of AML diagnosis was 12.7 months.

## Discussion

We describe the outcomes of a large real-world cohort of patients treated with single-agent gilteritinib for relapsed or refractory *FLT3*-mutated AML across the UK NHS. This cohort includes a significant proportion of patients with prior FLT3 inhibitor therapy, as well as the first described outcomes for patients receiving gilteritinib after venetoclax-based low-intensity therapy. Despite a lower remission rate, OS in this real-world population was similar to that seen in the ADMIRAL study.^8^

The characteristics of patients in our cohort were similar to those in ADMIRAL, apart from a higher proportion of patients with >1 line of therapy, prior FLT3 inhibitor, or prior venetoclax.^8^ Despite these features, which might be expected to be associated with poorer outcomes, the day 30 mortality (1% in our cohort vs 2% in ADMIRAL), number of cycles delivered (median 4 vs 5), proportion of patients who received transplantation (24% vs 26%), median OS (9.5 vs 9.3 months), and 1-year OS (38% vs 37%) were remarkably similar to the gilteritinib arm of ADMIRAL. These results provide reassurance that similar outcomes can be achieved in heavily pretreated real-world patients, many of whom may not have met trial eligibility criteria.

Although the survival outcomes were similar, the composite remission rate in our study, assigned according to ELN 2017 (CR + CRi 30%), was significantly lower than that in ADMIRAL in which a modification of the International Working Group criteria was applied (CR + CRi + CR with incomplete platelet recovery, 54%).^17^^,^^20^ Although it is difficult to directly compare these because of the different criteria applied, the lower rates we describe may be because of less frequent bone marrow biopsies and retrospective assessment of response by investigators in our study, and the inclusion of post-transplant remissions in the ADMIRAL study. However, we note that the proportion of patients with full CR (21% vs 21%) and overall response (including MLFS and PR, 68% vs 68%) was almost identical, suggesting that most of the observed difference is because of the criteria used.

Previous real-world studies have demonstrated similar outcomes to those we describe. A French series, which included 140 patients receiving single-agent gilteritinib (48% with prior midostaurin), reported CR/CRi (using ELN criteria) in 25.4% and median OS of 6.4 months.^11^ Another series from 11 US cancer centers included 71 patients receiving gilteritinib monotherapy, all of whom had prior FLT3i exposure, and using modified International Working Group criteria observed a cCR rate of 43%.^13^ OS was 7 months in the whole cohort, but this included patients receiving combination therapy. In both of these studies prior venetoclax exposure was not specified. Finally, in a multicenter series of 25 patients from Israel, of whom 8 had previously received FLT3i and 5 venetoclax, 48% achieved CR by ELN criteria and median OS was 6.4 months.^12^ Given the lack of previous reported data, we highlight the poor outcomes in our patients who were treated with gilteritinib after venetoclax-based front-line therapies, with median OS of 4.5 months and no patient alive at 18 months.

We noted a significantly lower remission rate in patients with FLT3-*TKD* mutations, with CR/CRi in only 9% of 22 patients. Although this was not seen in the ADMIRAL study and may be a chance finding due to small numbers, the French real-world study noted similar results, with CR/CRi of 15% in patients with *FLT3*-TKD only, compared with 29% in patients with ITD.^11^ The other group with particularly poor response rates was patients with adverse cytogenetics, with no responses seen in those with complex cytogenetics; *KMT2A* rearrangements; or abnormalities of chromosomes 5q, 7, and 17p. These findings were again mirrored in the French study with a 0% response rate in patients with adverse cytogenetics, and in the ADMIRAL study in which the HR for death in this subgroup was 1.63 (95% CI, 0.69-3.85). Studies investigating alternative strategies for these patients are required. Finally, we noted a lower response rate in those treated with gilteritinib after only 1 prior line of therapy, which we hypothesize may be because of different patient characteristics and an overrepresentation of *FLT3*-TKD in these patients.

A major advantage of gilteritinib as compared with intensive chemotherapy is the ability to deliver care in the outpatient setting. The ADMIRAL study did not report detailed information on duration of cytopenias or health care resource use. In this study, we found that a significant proportion of patients required hospital admission during the first 4 cycles, although for a duration shorter than would be expected for intensive chemotherapy. Although transfusion requirements were modest, they persisted throughout therapy, without a noticeable decrease in the number of patients requiring red cell or platelet transfusion across cycles 1 to 4. Our results reinforce the need for frequent monitoring and vigilant supportive care in these patients.

We acknowledge a number of limitations to this analysis, in particular the retrospective nature of the study, which introduces potential selection bias. We requested that sites query their departmental and pharmacy records to identify and include all potentially eligible patients, thereby limiting the selection bias. Responses were assigned by treating clinicians and investigators collecting data, and many patients did not have regular bone marrow biopsies, potentially limiting the utility of the response data. Finally, supportive care and toxicity data were only provided for a subset of the patients, which could introduce reporting bias.

In this real-world analysis of patients with relapsed or refractory *FLT3*-mutated AML treated with single-agent gilteritinib, outcomes mirrored those seen in the registration trial. However, outcomes for this group of patients remain disappointing, and further trials exploring alternative strategies are required. Our data provide a benchmark regarding response rate, toxicity, health care resource use, and long-term outcomes, which we hope will be useful in designing such studies.

Conflict-of-interest disclosure: J. Othman declares honoraria from Astellas and Jazz Pharmaceuticals. A.H. declares honoraria from Kite/Gilead. P.G. declares honoraria from Astellas. R. Dang declares meeting sponsorship from Jazz; and honoraria from AbbVie. J.V. declares meeting support from BeiGene, Janssen, and Jazz; and reports honoraria from AbbVie and AstraZeneca. P.K. declares honoraria from Jazz, Astellas, and Gilead; reports speakers bureau role with Astellas; and reports consultancy for Jazz and Gilead. A.-L.L. declares honoraria from Astella, AbbVie, Amgen, Kite, Novartis, Jazz, and Daiichi Sankyo; and speakers bureau role with Kite, Takeda, and Astellas. F.H. declares meeting sponsorship and honoraria from AbbVie. J.L. declares honoraria from Aptitude health. N.F. declares investigator meetings with Novartis and MEI Pharma. J. O’nions declares honoraria from Servier, Astellas, AbbVie, Jazz, and Janssen. Anjum Khan declares meeting sponsorship from Jazz, Medac, and Servier; speakers bureau role with AbbVie, Astellas, Jazz, and Servier; and consultancy/advisory board role for TC BioPharm, Incyte, Immedica, Novartis, Synairgen, and Takeda. R. Dillon declares research funding from AbbVie and Amgen; and consultancy with Astellas, Pfizer, Novartis, Jazz, BeiGene, Shattuck, and AvenCell. The remaining authors declare no competing financial interests.
