# Supplementary material for: Outcomes with single-agent gilteritinib for relapsed or refractory FLT3-mutant AML after contemporary induction therapy
Source: Blood Adv. 2024 Sep 16;8(21):5590–7. doi: 10.1182/bloodadvances.2024014017 (PMC11541456; doi:10.1182/bloodadvances.2024014017)

Outcomes with single-agent gilteritinib for relapsed or refractory *FLT3*-mutant AML after contemporary induction therapy

Supplemental appendix

Table of Contents

Table of Contents 2
List of centres and contributing clinicians 3
Table S1 – Comparison of patient characteristics with gilteritinib arm of ADMIRAL study 4
Table S2 – cytopenias and supportive care requirements in first 4 cycles 5
Table S3 – outcomes in *FLT3*-ITD AML by combination of *NPM1* and *DNMT3A* mutations 6
Table S4 – characteristics of patients with PR or better who did and did not proceed to transplant 7
Table S5 – gilteritinib as first salvage therapy after venetoclax and azacitidine/LDAC 8
Figure S1 – number of patients per site 9
Figure S2 – cumulative incidence of relapse/progression 10
Figure S3 – overall survival by best response 11
Figure S4 – outcomes in *FLT3*-ITD AML by combination of *NPM1* and *DNMT3A* mutations 12
Figure S5 – overall survival from allogeneic transplant 13

List of centres and contributing clinicians

Barts Cancer Institute - Paolo Gallipoli, Michael Austin, Kayleigh McCloskey, Matthew Smith, Michael Hamblin; Royal United Hospital Bath - Sally Moore, Laura Anderson; Queen Elizabeth Hospital Birmingham - Charles Craddock, Justin Loke; Birmingham Heartlands Hospital - Vidhya Murthy; Blackpool Hospital - Seye Kolade, Thishakya Wickramaratne, Asra Khan, Usman Afzal; Royal Sussex County Hospital - Tom Rider, Herng Mak; Bristol Royal Infirmary - Priyanka Mehta, Kathryn Fleming, Joe Cross; Addenbrooke's Hospital - Faisal Basheer, Charles Crawley, Shahzad Orthi, Jayalal Jayalal, Lee Mynott; University Hospital of Wales - Steven Knapper, Francesca Hogan, Victoria Ware; The Christie Hospital - Mike Dennis, Johnathan Elliot; University Hospital Coventry & Warwickshire - Duncan Murray, Beth Harrison, Martin Dyson, Jacob Thanakamma; Royal Derby Hospital - Ian Amott, Jamila Bashir; Kent and Canterbury Hospital - Sreetharan Munisamy; Frimley Park Hospital - Sofia Galli; Gloucestershire Royal Hospital - Adam Rye; Great Western Hospital - Alex Sternberg; Guy's Hospital - Richard Dillon, Jad Othman, Jamie Saunders, Ruebina Amofa; Basingstoke and North Hampshire Hospital - Henna Wong, Sylwia Simpson, Katherine Smith, Saniya Dhawan; Hull and East Yorkshire Hospitals - Simone Green, Mayanka Narayanan; Hammersmith Hospital - Renuka Palanicawandar; Ipswich Hospital - Mahesh Prahladan, Ioana Whalley; James Cook University Hospital - Raymond Dang, Oghenemaro Okah Avae, Georgina Clarke; King's College Hospital - Pramila Krishnamurthy, Jennifer Vidler; Kingston Hospital - Stefania Bonetto; St James' Cancer Institute - Anjum Khan, Manish Jain, Islam Abdallah, Chun Huat Teh, Richard Kelly; University Hospitals of Leicester - Katherine Hodgson, Alex Bashford; Lincoln County Hospital - Charlotte Kallmeyer; Norfolk and Norwich University Hospital - Angela Collins; University Hospitals of North Midlands - Srinivas Pillai; Peterborough City Hospital - Sateesh Nagumantry; Northwick Park Hospital - Vaitsa Katsomitrou, Hasan Jamjoom; Nottingham University Hospitals - Jennifer Byrne, Thomas Taylor, Denise Badder; Royal Oldham Hospital - David Osborne, Odong Ochaya; Derriford Hospital - Patrick Medd, Francesca Crolla; Queen Alexandra Hospital, Portsmouth - Edward Belsham, Behnaz Mobashwera; Queen Elizabeth University Hospital - Anne-Louise Latif, Cara Manson; Queen's Hospital Romford - Abbas Zaidi, Shabnam Banerjee, Jesca Boot; Royal Cornwall Hospital - David Tucker, Nicole Fowler, Claudia Bedford; Royal Devon and Exeter Hospital - Tom Coats; Royal Marsden Hospital - David Taussig, Madhu Sivarajah; Royal Surrey County Hospital - Elisabeth Grey-Davies; Royal Wolverhampton Hospital - Richard Whitmill; Royal Hallamshire Hospital - Alex Kanellopoulos; Royal Shrewsbury Hospital - George Cherian, Sarah Lane; Musgrove Park Hospital - Deepak Mannari, Francesca Crolla, Jayne Foot, Elizabeth Firth; Sunderland Royal Hospital - Scott Marshall, Emily Sutherland; University Hospital Southampton - Chris Dalley, Mariam Amer; Southend University Hospital - Pallavi Kalkur; Whiston Hospital - Eleana Loizou, Maryam Shahidianakbar, Emma Livesey, Dominika Radzova; Sandwell General Hospital - Farooq Wandroo; Torbay Hospital - Rui Zhao; University College Hospital - Jenny O'nions, Angela Hwang, Max Brodermann, Asim Khwaja; Western General Hospital - Victoria Campbell; Worthing Hospital - John Laurie, Heba Yassin

Table S1 – Comparison of patient characteristics with gilteritinib arm of ADMIRAL study

**Patient characteristics**	**UK real world N = 152**	**ADMIRAL N = 247**	**p-value***
Median age (range)	61 (19 – 90)	62 (20 – 84)	
Female	72 (47%)	131 (53%)	0.27
Performance status			
0 – 1	93 (82%)	206 (83%)	0.67
≥2	21 (18%)	41 (17%)	
Disease status			
Refractory to last line of therapy	53 (35%)	98 (40%)	0.34
Relapse	99 (65%)	149 (60%)	
Number of prior lines therapy			
One prior line	98 (64%)	247 (100%)	<0.01
Two or more	54 (36%)	0 (0%)	
Previous therapies			
FLT3 inhibitor*	63 (41%)	34 (14%)	<0.01
Midostaurin	58 (38%)	21 (5.7%)	<0.01
Quizartinib	3 (2.0%)	0 (0%)	0.05
Sorafenib	7 (4.6%)	13 (5.3%)	0.82
Intensive chemotherapy	121 (80%)	205 (83%)	0.42
Venetoclax	37 (24%)	Not reported	<0.01
Allogeneic transplant	29 (19%)	48 (19.4)	1.0
FLT3-ITD*	134 (88%)	217 (88%)	1.0
FLT3-TKD*	23 (16%)	23 (9.3%)	0.11
MRC cytogenetic risk			
Favourable	0	4 (1.6%)	0.58
Intermediate	134 (88%)	182 (74%)	
Adverse	18 (12%)	26 (11%)	
NPM1 mutation	52 (34%)	115 (48%)	0.02
Molecular mutations^			
DNMT3A	44 (35%)	75 (31%)	0.48
IDH1/2	21 (26%)	38 (16%)	0.88
WT1	12 (9.6%)	45 (19%)	0.02
Missing	27	8	

*By Fisher's exact test

^Proportion of assessable patients (125 in our cohort and 239 in ADMIRAL)

Table S2 – cytopenias and supportive care requirements in first 4 cycles

Characteristic	Cycle 1	Cycle 2	Cycle 3	Cycle 4
**Number with data recorded for cycle**	**87**	**77**	**56**	**44**
Days of grade 4 neutropenia, median (IQR)	18 (1, 28)	10 (0, 28)	7 (0, 28)	4 (0, 27)
Days of grade 4 thrombocytopenia, median (IQR)	4 (0, 20)	6 (0, 17)	0 (0, 16)	0 (0, 16)
Proportion with any hospital admission	49 (56%)	32 (42%)	15 (27%)	16 (36%)
Median days in hospital (IQR)*	10 (4, 22)	13 (8, 22)	4 (3, 16)	9 (6, 14)
Proportion with any ICU admission	3 (3.4%)	1 (1.3%)	0 (0%)	1 (2.3%)
Median days in ICU (IQR)^	5 (4, 7)	2 (2, 2)	0 (0, 0)	2 (2, 2)
Required IV antibiotics<br>*Data not available*	35 (43%)<br>*5*	30 (39%)<br>*0*	9 (16%)<br>*0*	10 (23%)<br>*0*
Median days on IV antibiotics (IQR)⁺	8 (5, 13)	12 (7, 19)	3 (3, 7)	2 (1, 5)
Required red blood cell transfusion<br>*Data not available*	52 (68%)<br>*10*	49 (71%)<br>*7*	29 (56%)<br>*4*	23 (59%)<br>*5*
Median number RBC units transfused (IQR)**	2 (0, 4)	2 (0, 5)	2 (0, 5)	2 (0, 4)
Required platelet transfusion<br>*Data not available*	38 (49%)<br>*10*	38 (54%)<br>*7*	24 (46%)<br>*4*	20 (51%)<br>*5*
Median number platelet units transfused (IQR)**	0 (0, 3)	1 (0, 5)	0 (0, 5)	1 (0, 4)

*Including only patients requiring hospitalisation
^ Including only patients requiring ICU admission
⁺ Including only patients requiring IV antibiotics
**Includes all patients

Table S3 – outcomes in *FLT3*-ITD AML by combination of *NPM1* and *DNMT3A* mutations

Characteristic	Both wild-type	*DNMT3A* mutated	*NPM1* mutated	Both mutated	P value
Number	55	18	14	21	
CR/CRi	18 (35%)	4 (22%)	2 (15%)	7 (35%)	0.5
12-month OS	46%	35%	24%	39%	0.3
Median OS	11.1	7.7	4.0	9.4	

Table S4 – characteristics of patients with PR or better who did and did not proceed to transplant

**Characteristic**	**All**<br>N = 98	**No alloSCT**<br>N = 73	**AlloSCT**<br>N = 25	**p-value**
Median age (range)	61 (51, 73)	69 (57, 74)	44 (33, 57)	<0.001
Female	51 (52%)	41 (56%)	10 (40%)	0.2
Performance status				0.013
0 – 1	53 (80%)	34 (72%)	19 (100%)	
≥2	13 (20%)	13 (28%)	0 (0%)	
Missing	32	26	6	
Clinical disease type				0.7
De novo	75 (77%)	54 (74%)	21 (84%)	
Secondary	19 (19%)	15 (21%)	4 (16%)	
Therapy-related	4 (4.1%)	4 (5.5%)	0 (0%)	
Disease status				0.004
Refractory to last line of therapy	32 (33%)	18 (25%)	14 (56%)	
Relapse	66 (67%)	55 (75%)	11 (44%)	
Number of prior lines therapy (range)				0.3
One prior line	59 (60%)	46 (63%)	13 (52%)	
Two or more	39 (40%)	27 (37%)	12 (48%)	
Prior FLT3 inhibitor	40 (41%)	25 (34%)	15 (60%)	0.024
Prior venetoclax	24 (24%)	19 (26%)	5 (20%)	0.5
Intensity of first line AML therapy				0.15
Intensive chemotherapy	76 (78%)	54 (74%)	22 (88%)	
Low intensity	22 (22%)	19 (26%)	3 (12%)	
MRC cytogenetic risk				0.7
Intermediate	91 (93%)	67 (92%)	24 (96%)	
Adverse	7 (7.1%)	6 (8.2%)	1 (4.0%)	
FLT3-ITD	92 (94%)	68 (93%)	24 (96%)	>0.9
FLT3-TKD	7 (7.3%)	6 (8.3%)	1 (4.2%)	0.7
ELN 2022 risk classification				0.6
Favourable	1 (1.0%)	1 (1.4%)	0 (0%)	
Intermediate	66 (67%)	47 (64%)	19 (76%)	
Adverse	31 (32%)	25 (34%)	6 (24%)	

Table S5 – gilteritinib as first salvage therapy after venetoclax and azacitidine/LDAC

**Characteristic**	**N = 20**
Median age (range)	72.2 (54-90)
Female	12 (60%)
Performance status	
0 – 1	9 (69%)
≥2	4 (31%)
Missing	7
Clinical disease type	
De novo	13 (65%)
Secondary	7 (35%)
Therapy-related	0
Disease status	
Refractory to last line of therapy	7 (35%)
Relapse	13 (65%)
Prior FLT3 inhibitor	0 (0%)
Previous allogeneic transplant	0 (0%)
MRC cytogenetic risk	
Intermediate	18 (90%)
Adverse	2 (10%)
FLT3-ITD	17 (85%)
FLT3-TKD	4 (20%)
Evolution of *FLT3* mutation	
Present at diagnosis and relapse	12 (60%)
Emergent upon relapse	8 (40%)
NPM1 mutation	7 (35%)
ELN 2022 risk classification	
Favourable	1 (5.0%)
Intermediate	9 (45%)
Adverse	10 (50%)
**Outcomes**	
Best response to gilteritinib	
Complete remission	2 (10%)
Complete remission with incomplete hematologic recovery	3 (15%)
Morphologic leukemia-free state	1 (5.0%)
Partial remission	7 (35%)
Refractory disease	6 (30%)
Death prior to response assessment	1 (5.0%)
Median OS (months)	4.5
12-month OS	15%
Median OS from AML diagnosis (months)	12.7

Figure S1 – number of patients per site

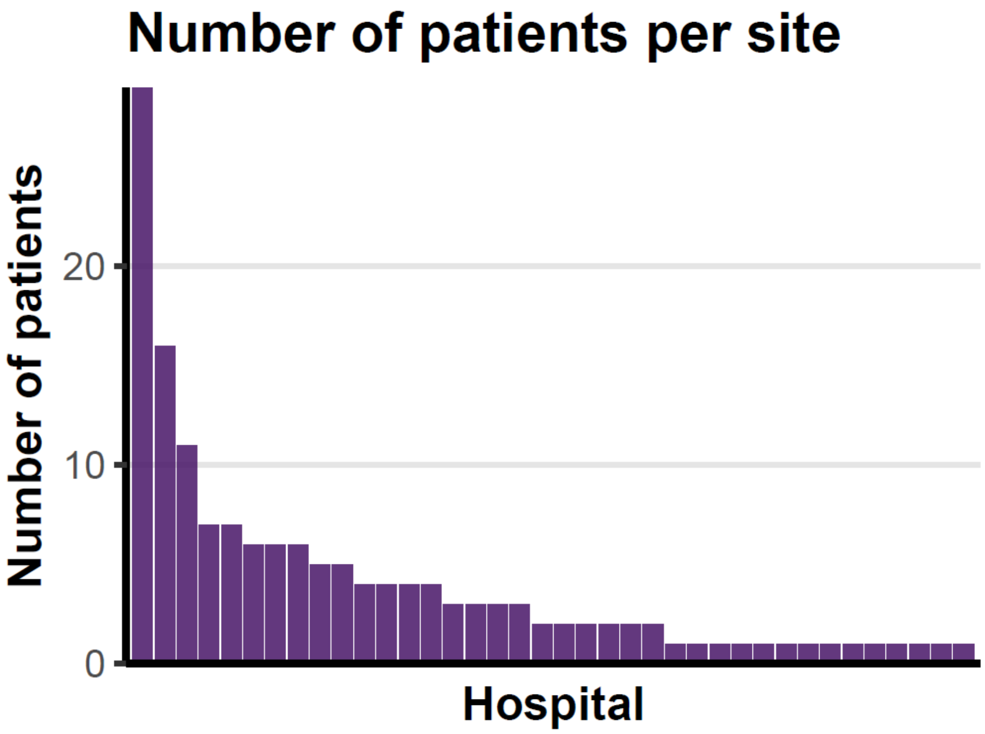

Figure S2 – cumulative incidence of relapse/progression

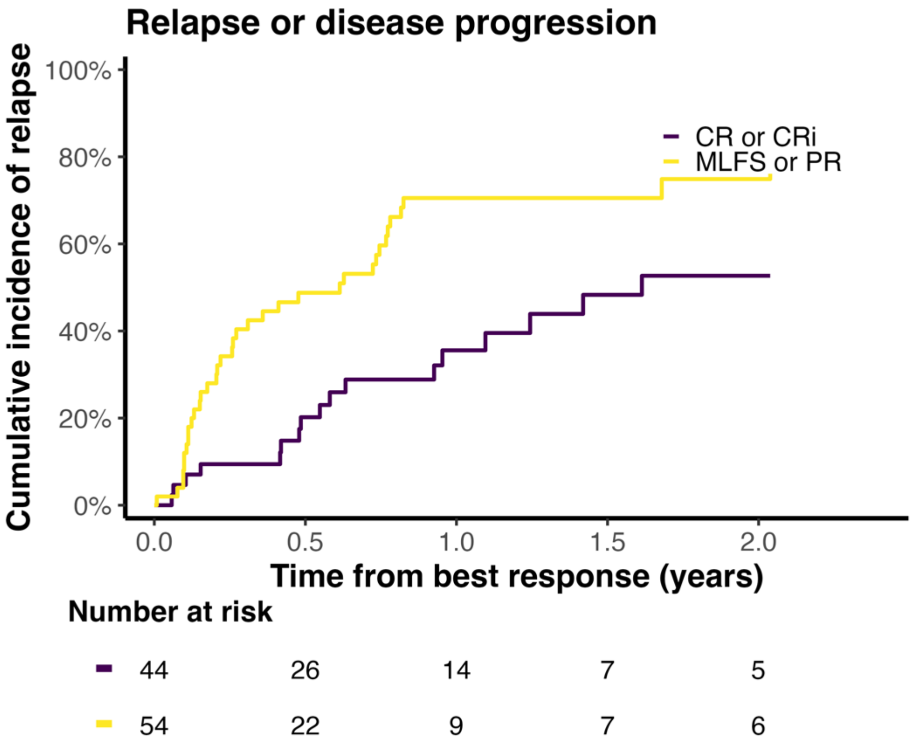

Figure S3 – overall survival by best response

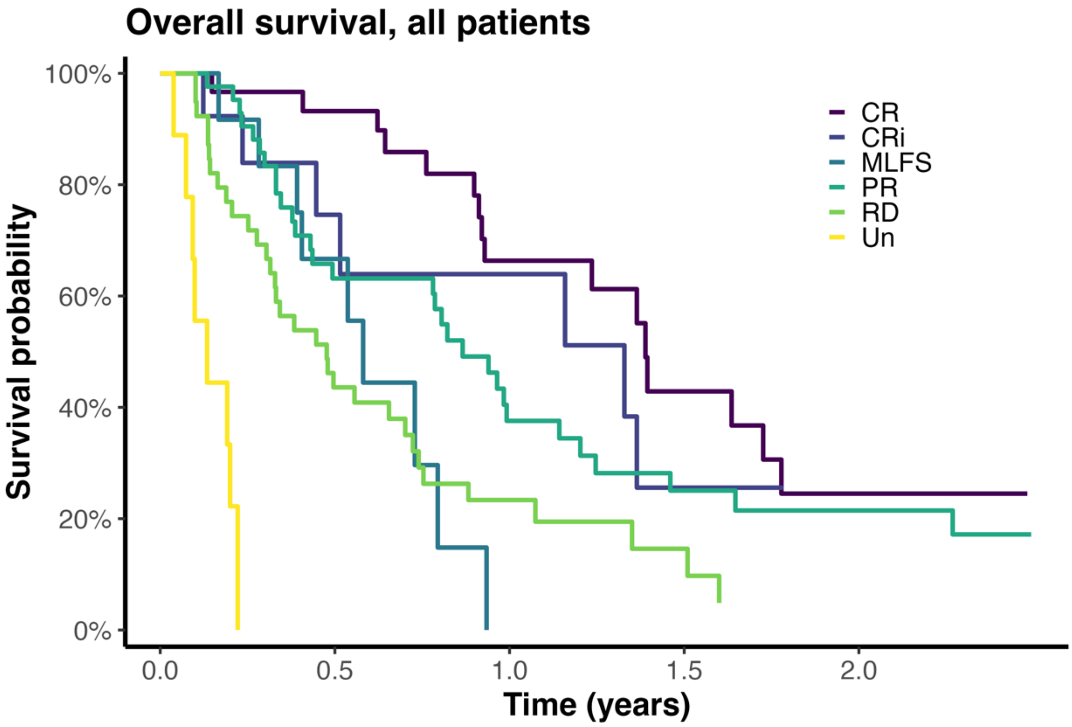

Number at risk

31	27	17	7	4
13	7	6	2	1
12	6	0	0	0
42	24	13	8	5
39	17	8	3	1
9	0	0	0	0

Figure S4 – outcomes in *FLT3*-ITD AML by combination of *NPM1* and *DNMT3A* mutations

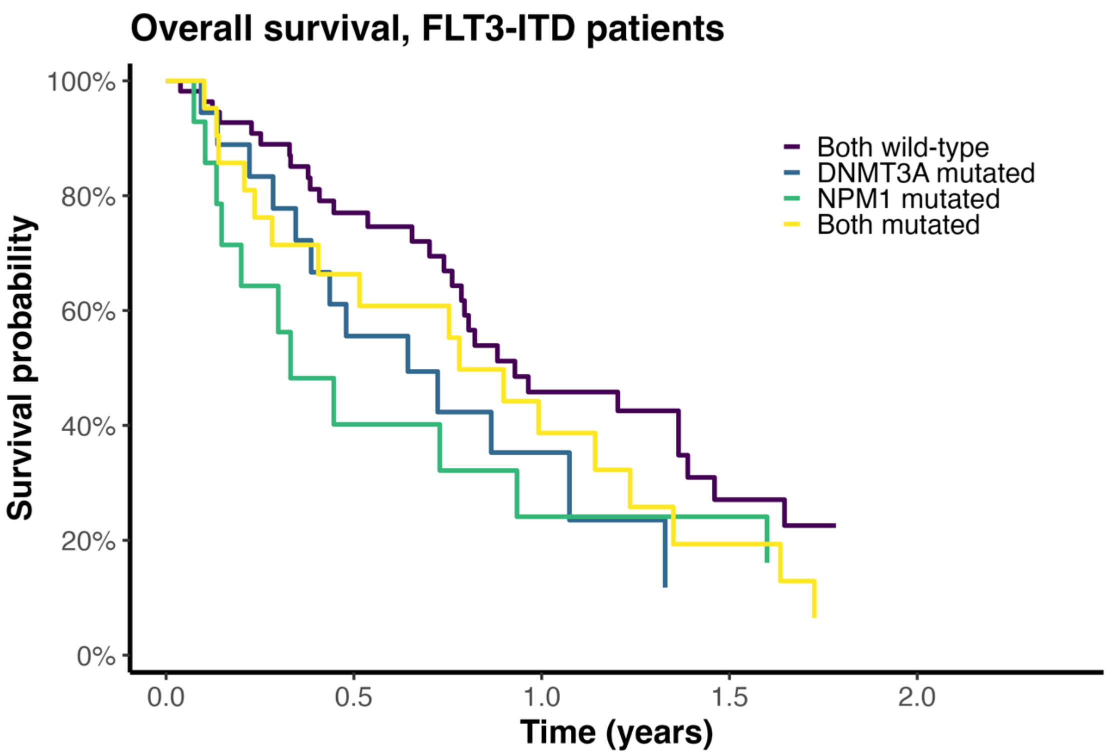

Number at risk

	0.0	0.5	1.0	1.5	2.0
Both wild-type	55	34	17	7	3
DNMT3A mutated	18	10	5	1	1
NPM1 mutated	14	5	3	3	2
Both mutated	21	12	7	3	1

Figure S5 – overall survival from allogeneic transplant

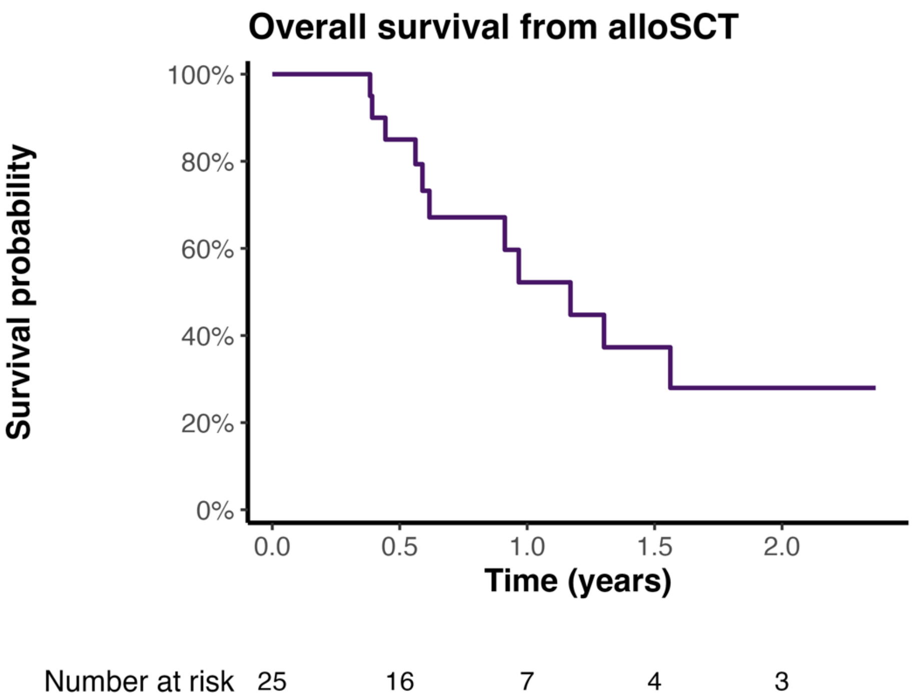

Supplement: Supplemental Appendix, Tables, and Figures [file BLOODA_ADV-2024-014017-mmc1.pdf]
